# Modifiable lifestyle factors have a larger contribution to colorectal neoplasms than family history

**DOI:** 10.1186/s12885-022-10141-1

**Published:** 2022-10-07

**Authors:** Shuyuan Wang, Zhen Yuan, Yuqi Wang, Xuanzhu Zhao, Weifeng Gao, Hongzhou Li, Yuanshun Zhao, Zili Zhang, Shuiqing Liang, Zhaoce Liu, Qinghuai Zhang, Hong Ma, Xipeng Zhang, Wei Cui, Chunze Zhang

**Affiliations:** 1 School of Medicine, Nankai University, Tianjin, China; 2 Department of Colorectal Surgery, Tianjin Union Medical Center, Tianjin, China; 3 School of Integrative Medicine, Tianjin University of Traditional Chinese Medicine, Tianjin, China; 4 Department of Endoscopy, Tianjin Union Medical Center, Tianjin, China; 5 Tianjin Third Central Hospital, Tianjin, China; 6 Dagang Hospital of Tianjin Binhai New Area, Tianjin, China; 7 Tianjin Institute of Coloproctology, Tianjin, China; 8 The Institute of Translational Medicine, Tianjin Union Medical Center of Nankai University, Tianjin, China; 9 Department of Nursing, Tianjin Union Medical Center, Tianjin, China; 10 School of Mathematical Sciences and LPMC, Nankai University, Tianjin, China

**Keywords:** Colorectal Neoplasms, Population Attributable Fraction, Risk Factors

## Abstract

**Background:**

Screening recommendations for colorectal cancer (CRC) are mainly based on family history rather than lifestyle risk factors. We aimed to assess and compare risk factors for colorectal neoplasm (CRN) and evaluate trends in neoplasm detection rates during the three rounds of screening from 2012 to 2020 in Tianjin, China.

**Methods:**

This study was based on 89,535 first-recorded colonoscopies in Tianjin CRC screening program, 2012–2020. Of these, 45,380 individuals with complete family history and lifestyle factors were included for population attributable fraction (PAF) estimation.

**Results:**

The overall detection rate of nonadvanced adenomas, advanced adenomas and CRC was 39.3%, 5.9% and 1.5%, respectively. The PAFs of current smoking, alcohol consumption, physical activity, higher BMI and family history of CRC, respectively, were 8.9%, 2.6%, 1.9%, 5.8%, and 1.1% for males with nonadvanced CRN; 12.3%, 7.3%, 4.9%, 7.2%, and 0.8% for males with advanced CRN; 3.4%, 0.4%, 2.1%, 7.8%, and 0.7% for females with nonadvanced CRN; and 4.3%, 0.2%, 8.2%, 8.5%, and -0.6% for females with advanced CRN. The PAFs of selected lifestyle factors were 19.9% for males with nonadvanced CRN, 29.0% for males with advanced CRN, 9.7% for females with nonadvanced CRN and 13.8% for females with advanced CRN.

**Conclusions:**

Modifiable lifestyle factors, including smoking, alcohol consumption, physical activity and BMI, have a larger contribution to CRN than family history of CRC. Our findings will provide references for developing guidelines of CRC prevention and control in China.

**Supplementary Information:**

The online version contains supplementary material available at 10.1186/s12885-022-10141-1.

## Background

Colorectal cancer (CRC) is the third-most common cancer among men and the second-most common cancer among women worldwide [1, 2]. Over the past decade, CRC screening has been implemented in several regions of China, carried out via a questionnaire survey, followed by the faecal occult blood test (FOBT) and colonoscopy [3, 4]. Similar to most countries, high-risk assessment in China is mainly based on family history and personal history of polyps, CRC or inherited CRC syndrome but does not take other risk factors into account. However, an increasing amount of evidence has suggested an essential role of lifestyle factors in the development of colorectal cancer [5–8], and these factors are potentially preventable.

Most prior studies assessing attributable causes of cancer have been conducted in high-resource countries, primarily Western countries [8–12]. As the pattern of cancer has wide regional and socioeconomic variation [13, 14], it would not be appropriate to apply the population attributable fraction (PAF) estimated on Western populations to other countries. Only a few studies assessing CRCs attributable to known risk factors have been conducted in China [15–17]; moreover, all of these studies were based on data collected at least a decade ago and did not consider the risk factors for a family history of CRC. However, China has witnessed rapid changes in socioeconomic status and lifestyle patterns over the past decade.

Thus, we conducted this cross-sectional study to assess the PAFs of modifiable lifestyle factors, including smoking, alcohol drinking, physical inactivity, body mass index (BMI), as well as family history of CRC in first-degree relatives (FDR).

## Methods

### Study population

This cross-sectional study was conducted using the database of individuals undergoing screening colonoscopy at medical institutions designated by the programme in Tianjin, China, between 2012 and 2020. The screening protocol was developed by Tianjin CRC Screening Office and was performed in a 3-year cycle, with the first round from 2012 to 2014, the second round from 2015 to 2017 and the third round from 2018 to 2020. Within each round, the main screening population for the first year is individuals aged 60–74 years old, for the second year is 50–60 years old and for the third year is 40–50 years old. The high-risk assessment was based on first-degree relative with CRC, a personal history of adenoma or polyps, the result of FOBT and intestinal symptoms [4, 18]. High-risk participants were highly recommended to undergo a colonoscopy examination through advice notes issued by the screening physicians and were followed up by the primary care units, while for nonhigh-risk participants, colonoscopies were voluntary.

A total of 89,535 first-recorded colonoscopies from both high-risk and nonhigh-risk participants were included in present study. Individuals with missing or unknown family history in FDR (*n* = 216), and those with incomplete lifestyle factors (*n* = 40,445), including smoking, alcohol consumption, physical activity and BMI, were excluded. Moreover, we also excluded individuals with a personal history of intestinal polyp or cancer (*n* = 3,494), because these patients might have negative or normal findings as a result of an earlier polypectomy. All participants provided written informed consent before enrollment, and all investigations and methods used were in accordance with the Declaration of Helsinki. This study was approved by the ethics committee of Tianjin Union Medicine Center.

### Measurements and definitions

All colonoscopies were performed by experienced endoscopists who had at least 5 years of experience and were all board certified to perform endoscopy in designated medical institutions. All abnormal findings were confirmed by expert gastrointestinal pathologists following up-to-date clinical guidelines. Only high-quality colonoscopies were included, with adequate bowel preparation, photo documentation of caecal landmarks, and a withdrawal time > 6 min.

In this study, we categorized colonoscopy findings into 3 groups: advanced CRN, nonadvanced CRN (equivalent to nonadvanced adenoma) and normal colonoscopy. Advanced CRN was defined as CRC or advanced adenoma ≥ 10 mm in diameter or with villous components or high-grade dysplasia. Normal colonoscopy referred to a colonoscopy in which no adenoma or CRC was found. Smoking status was categorized as never smoker, current smoker and former smoker. Alcohol consumption was categorized as never drinker and ever drinker, with the former including those never drinking and rarely drinking. The quantity of alcohol consumption per day was collected in a Chinese unit that 1 unit refers to 50 g Chinese spirits. In this study, the amount of alcohol consumed per each unit (50 g Chinese spirits) was calculated based on a common type of Chinese spirits (38°), which contains approximately 17 g alcohol in one unit. Physical activity was estimated by combining the frequency and the duration of each exercise. Regular activity was defined as more than 30 min of physical activity at least once per week; otherwise, it was classified as ‘physical inactivity’. Overweight was defined as 25 ≤ BMI < 30 kg/m^2^, and obesity was defined as BMI ≥ 30 kg/m^2^. Educational level was categorized as low (primary education or below), intermediate (secondary education, high education or lower vocational education) and high (higher vocational education, university or above).

### Statistical analysis

Strict standards were applied to ensure the quality of the screening data. The validity of questionnaire and data-entry was checked by trained study staff, with a consistency check was conducted. After the data were transmitted from primary care units to the Office of Tianjin Colorecta Cancer Screening Programme, it was checked again by Programme Office, and 4% of the preliminary data would be randomly selected for quality control and calling back interview.

The adjusted PAFs were calculated to describe the proportion of cases in the population that is attributable to the exposure. PAF here could be interpreted as the proportion of CRN cases that would not occur if individuals had no exposure to risk factors. The adjusted PAF estimation based on a logistic regression model is commonly used for cross-sectional or case–control sampling designs [19, 20]. The attributable fraction (AF) is defined as *AF* = 1 − (*Pr*(*Y* = 1)/*Pr*(*Y*0​ = 1)​), where *Pr*(*Y*0​ = 1) denotes the counterfactual probability of the outcome if the exposure would have been eliminated from the population and *Pr*(*Y* = 1) denotes the factual probability of the outcome; logistic regression was used to adjust confounders [20, 21]. The theory and estimation strategy were descripted in detail by Dahlqwist et al. [22]. Briefly, a logistic regression model is fitted to the data in the first step; then, for each subject *i* with covariate vector *Zi* the model is used to estimate OR^−*Xi*^(*Zi*). For exposed subjects (those with *Xi* = 1), OR^−*Xi*^(*Zi*) = OR^−1^(*Zi*) and for unexposed subjects (those with *Xi* = 0), OR^−*Xi*^(*Zi*) = 1; the predictions of OR^−*Xi*^(*Zi*) are then averaged among the cases (those with *Yi* = 1), to produce an estimate of AF [22]. The confounder-adjusted PAF estimation was calculated using the "AF" package in R [21].

Pearson chi-square tests were performed to compare the detection rate between males and females. A multivariable logistic regression model was performed to assess the association between risk factors and colorectal neoplasms; odds ratios (ORs) and 95% confidence intervals (CIs) were calculated. The model was adjusted for age, educational level, marital status, smoking status, alcohol consumption, physical activity, BMI, family history of CRC in FDR and year of colonoscopy. All analyses were performed using R software (V.4.1.2). Two-sided *P* values < 0.05 were considered to be statistically significant.

## Results

### Detection rates of colorectal neoplasms

Overall, there were 89,535 first-recorded and high-quality colonoscopies performed within the screening programme from 2012 to 2020 in Tianjin, China. Of these, 41994 (46.9%) were male and 47541 (53.1%) were female; the mean (SD) age was 64.3 (8.5) years. Among males, 3621 (8.6%) and 17839 (42.5%) were diagnosed with advanced and nonadvanced neoplasm, respectively; among females, 2211 (4.7%) and 14381 (30.2%) were diagnosed with advanced and nonadvanced CRN, respectively. The detection rate for advanced neoplasm in the first (2012–2014), second (2015–2017), and third (2018–2020) round was 5.9%, 5.7% and 8.4%, respectively (*p* < 0.001), while that for nonadvanced neoplasm was 24.1%, 35.6% and 41.7%, respectively (*p* < 0.001).

### Characteristics of the study population

A total of 45,380 individuals were included in assessing neoplasms attributable to lifestyle factors and family history, with the characteristics shown in Table 1. There were more females than males (F:M = 1.12:1) in the final analysis, and 44.4% of males and 61.2% of females had a normal colonoscopy (*p* < 0.001). The mean (SD) age was 62.9 (7.9) years, with 47.5% of them aged 60–70 years old. Only 13.7% of individuals had a high level of education, and 95.8% were married. Approximately half of them were overweight or obese. The proportions of current smokers and ever drinkers in males were significantly higher than those in females (33.0% vs. 5.7%; 21.0% vs. 0.8%, respectively, *p* < 0.001). Overall, 7.8% had a history of CRC in FDR, with 7.2% in males and 8.4% in females.

**Table 1:** Characteristics of 45,380 participants stratified by sex

	Total (*n* = 45,380)	Male (*n* = 21,415)	Female (*n* = 23,965)
**Age at colonoscopy, years (%)**			
< 50	2896 (6.4)	1469 (6.9)	1427 (6.0)
50–60	11,597 (25.6)	5054 (23.6)	6543 (27.3)
60–70	21,403 (47.2)	10,010 (46.7)	11,393 (47.5)
≥ 70	9484 (20.9)	4882 (22.8)	4602 (19.2)
**Educational level (%)**			
Low	9166 (20.2)	3784 (17.7)	5382 (22.5)
Intermediate	30,013 (66.1)	14,224 (66.4)	15,789 (65.9)
High	6201 (13.7)	3407 (15.9)	2794 (11.7)
**Marital status (%)**			
Married	43,468 (95.8)	20,814 (97.2)	22,654 (94.5)
Unmarried	429 (0.9)	218 (1.0)	211 (0.9)
Divorced	343 (0.8)	128 (0.6)	215 (0.9)
Widowed	1140 (2.5)	255 (1.2)	885 (3.7)
**Smoking status (%)**			
Never	35,007 (77.1)	12,703 (59.3)	22,304 (93.1)
Former	1928 (4.2)	1640 (7.7)	288 (1.2)
Current	8445 (18.6)	7072 (33.0)	1373 (5.7)
Cigarettes per day, mean (SD)	16.01 (9.30)	16.38 (9.51)	14.06 (7.81)
**Alcohol consumption (%)**			
Never drinker	40,699 (89.7)	16,915 (79.0)	23,784 (99.2)
Ever drinker	4681 (10.3)	4500 (21.0)	181 (0.8)
Alcohol (g) consumed per day^b^, mean (SD)	38.98 (28.53)	39.24 (24.77)	31.53 (80.85)
**Physical activity (%)**			
Physical inactivity	25,411 (56.0)	11,636 (54.3)	13,775 (57.5)
Regular activity	19,969 (44.0)	9779 (45.7)	10,190 (42.5)
**BMI, kg/m**^**2**^** (%)**			
< 25	26,124 (57.6)	11,865 (55.4)	14,259 (59.5)
25–29.9	16,414 (36.2)	8319 (38.8)	8095 (33.8)
≥ 30	2842 (6.3)	1231 (5.7)	1611 (6.7)
**Colonoscopy findings (%)**			
Normal	24,176 (53.3)	9501 (44.4)	14,675 (61.2)
Nonadvanced CRN	17,823 (39.3)	9788 (45.7)	8035 (33.5)
Advanced CRN	3381 (7.5)	2126 (9.9)	1255 (5.2)
Advanced adenoma	2688 (79.5)	1714 (80.6)	974 (77.6)
CRC	693 (20.5)	412 (19.4)	281 (22.4)
**Family history of CRC in FDR (%)**			
No	41,820 (92.2)	19,863 (92.8)	21,957 (91.6)
Yes	3560 (7.8)	1552 (7.2)	2008 (8.4)
**Tumor location**^**a**^** (%)**			
Colon	14,524 (68.5)	7964 (66.8)	6560 (70.6)
Rectum	2867 (13.5)	1494 (12.5)	1373 (14.8)
Unspecific	3813 (18.0)	2456 (20.6)	1357 (14.6)

*CRN* Colorectal neoplasm, *BMI* Body mass index, *CRC* Colorectal cancer, *FDR* First-degree relative

^a^ The distribution of tumor location was based on 21,204 participants who diagnosed with colorectal neoplasms

^b^ The amount of consumed alcohol was calculated based on a common type of Chinese spirits (38°), which contains about 17.1 g alcohol in one unit (50 g spirits)

### Association of risk factors with colorectal neoplasms

Current smoking, alcohol drinking and overweight or obese were significantly associated with an increased odds of nonadvanced CRN in both males and females, whereas former smoking and physical inactivity had no significant association with nonadvanced CRN (Table 2). Males with a history of CRC in FDR had a 1.13-fold increased risk of nonadvanced CRN, while females had a nonsignificant 1.08-fold increased risk of nonadvanced CRN. Moreover, no significant association was found between the intensity of smoking (number of cigarettes per day) or drinking (grams of alcohol consumption per day) with advanced neoplasm. In the current-smoking subgroup, the OR for each additional cigarette per day was 1.01 (95%CI = 0.99–1.02, *p* = 0.052) in males and 0.99 (95%CI = 0.97–1.02, *p* = 0.595) in females; in the drinking subgroup, the OR for each additional unit of Chinese spirit per day was 1.03 (95%CI = 0.97–1.10, *p* = 0.315) in males and 0.81 (95%CI = 0.20–3.37, *p* = 0.777) in females. Additional logistic regressions with smoking and drinking categorized by intensity were shown in Table S1.

**Table 2:** Association of selected risk factors with colorectal neoplasms stratified by sex

	Male			Female		
	Number of cases with risk factor (%)	Adjusted OR^a^ (95% CI)	*P*	Number of cases with risk factor (%)	Adjusted OR^a^ (95% CI)	*P*
**For nonadvanced CRN**						
Smoking status (vs. never)						
Former	743 (7.6)	0.93 (0.83–1.04)	0.233	110 (1.4)	1.12 (0.87–1.44)	0.379
Current	3484 (35.6)	1.31 (1.22–1.40)	< 0.001	630 (7.8)	1.79 (1.59–2.02)	< 0.001
Alcohol consumption (vs. never)						
Ever drinker	2190 (22.4)	1.14 (1.06–1.23)	< 0.001	88 (1.1)	1.54 (1.13–2.10)	0.007
Physical activity (vs. regular activity)						
Physical inactivity	5241 (53.5)	1.01 (0.95–1.08)	0.665	4512 (56.2)	1.02 (0.96–1.08)	0.542
BMI, kg/m^2^ (vs. < 25)						
25–29.9	3916 (40.0)	1.12 (1.05–1.19)	< 0.001	2916 (36.3)	1.19 (1.12–1.26)	< 0.001
≥ 30	624 (6.4)	1.36 (1.19–1.54)	< 0.001	630 (7.8)	1.36 (1.22–1.52)	< 0.001
Family history of CRC in FDR (vs. no)						
Yes	757 (7.7)	1.13 (1.01–1.27)	0.026	707 (8.8)	1.08 (0.98–1.19)	0.134
**For advanced CRN**						
Smoking status (vs. never)						
Former	156 (7.3)	0.84 (0.70–1.02)	0.083	22 (1.8)	1.38 (0.87–2.19)	0.174
Current	821 (38.6)	1.42 (1.27–1.58)	< 0.001	112 (8.9)	2.05 (1.64–2.57)	< 0.001
Alcohol consumption (vs. never)						
Ever drinker	574 (27.0)	1.41 (1.25–1.59)	< 0.001	12 (1.0)	1.20 (0.64–2.25)	0.565
Physical activity (vs. regular activity)						
Physical inactivity	1089 (51.2)	1.05 (0.95–1.16)	0.346	697 (55.5)	1.11 (0.98–1.26)	0.094
BMI, kg/m^2^ (vs. < 25)						
25–29.9	869 (40.9)	1.15 (1.04–1.28)	0.006	457 (36.4)	1.20 (1.06–1.36)	0.005
≥ 30	148 (7.0)	1.49 (1.21–1.82)	< 0.001	112 (8.9)	1.54 (1.24–1.92)	< 0.001
Family history of CRC in FDR (vs. no)						
Yes	151 (7.1)	1.13 (0.93–1.36)	0.221	92 (7.3)	0.92 (0.74–1.16)	0.484

*CRN* Colorectal neoplasm, *OR* Odds ratio, *CI* Confidence interval, *BMI* Body mass index, *CRC* Colorectal cancer, *FDR* First-degree relative

^a^ ORs were adjusted for age at colonoscopy, educational level, marital status, smoking status, alcohol intake, physical activity, BMI, family history of CRC in first-degree relative and year of colonoscopy

The risk factors for advanced CRN were similar to those for nonadvanced CRN and were even stronger in both males and females (Table 2). Family history of CRC in FDR, unexpectedly, was not significantly associated with increased odds in either sex.

### The population attributable fractions of risk factors

Table 3 shows the PAFs and the number of cases attributable to risk factors. The highest PAFs of nonadvanced and advanced CRN in males were for current smoking (8.9% and 12.3%, respectively), followed by high BMI (5.8% and 7.2%, respectively) and alcohol drinking (2.6% and 7.3%, respectively); in females, PAFs were highest for high BMI (7.8% and 8.5%, respectively), followed by current smoking (3.4% and 4.3%, respectively) and physical inactivity (2.1% and 8.2%, respectively), and negligible for alcohol drinking (0.4% and 0.2%, respectively). PAFs of nonadvanced and advanced CRN associated with a history of CRC in FDR were relatively low in both sexes (1.1% and 0.7% in males; 0.8% and -0.6% in females).

**Table 3:** Number and PAFs of colorectal neoplasms attributable to selected risk factors

	Male		Female	
	Adjusted PAF^a^ (95% CI)	Cases attributable to risk factor^b^	Adjusted PAF^a^ (95% CI)	Cases attributable to risk factor^b^
**For nonadvanced CRN**				
Current smoking	8.9% (6.6% to 11.1%)	871/9788	3.4% (1.6% to 5.2%)	273/8035
Ever drinking	2.6% (0.6% to 4.6%)	254/9788	0.4% (-1.4% to 2.2%)	32/8035
Physical inactivity	1.9% (-1.6% to 5.3%)	186/9788	2.1% (-1.5% to 5.7%)	169/8035
BMI > 25 kg/m^2^	5.8% (3.1% to 8.5%)	567/9788	7.8% (5.1% to 10.4%)	627/8035
History of CRC in FDR	1.1% (-0.5% to 2.7%)	108/9788	0.7% (-1.2% to 2.7%)	56/8035
All selected lifestyle risk factors^c^	19.9% (14.4% to 25.3%)	1948/9788	9.7% (5.1% to 14.2%)	779/8035
**For advanced CRN**				
Current smoking	12.3% (7.9% to 16.8%)	261/2126	4.3% (-0.8% to 9.5%)	54/1255
Ever drinking	7.3% (3.0% to 11.5%)	155/2126	0.2% (-5.1% to 5.5%)	3/1255
Physical inactivity	4.9% (-1.1% to 10.9%)	104/2126	8.2% (0.6% to 15.8%)	103/1255
BMI > 25 kg/m^2^	7.2% (1.8% to 12.6%)	153/2126	8.5% (1.9% to 15.1%)	107/1255
History of CRC in FDR	0.8% (-3.2% to 4.8%)	17/2126	-0.6% (-6.2% to 5%)	8/1255
All selected lifestyle risk factors^c^	29.0% (20.4% to 37.6%)	617/2126	13.8% (4.2% to 23.3%)	173/1255

*PAF* Population attributable fraction, *CI* Confidence interval, *CRN* Colorectal neoplasm, *BMI* Body mass index, *CRC* Colorectal cancer, *FDR* First-degree relative

^a^ PAFs were adjusted for age at colonoscopy, educational level, marital status, smoking status, alcohol intake, physical activity, BMI, family history of CRC in first-degree relative and year of colonoscopy

^b^ [Total number of cases × PAF] / total number of cases

^c^ Selected lifestyle risk factors including current smoking, alcohol drinking, physical inactivity and BMI > 25 kg/m^2^

The PAFs for all of the selected lifestyle risk factors (not including family history of CRC) considered in this study were 19.9% for nonadvanced and 29.0% for advanced CRN in males and 9.7% and 13.8% in females, respectively.

## Discussion

In this large cross-sectional study with 45,380 participants who underwent colonoscopy within the CRC screening program in Tianjin, China, from 2012 to 2020, a total of 19.9% of nonadvanced and 29.0% of advanced CRNs in males and 9.7% of nonadvanced and 13.8% of advanced CRNs in females were attributable to modifiable lifestyle factors, including current, smoking, alcohol drinking, high BMI and physical inactivity, but only approximately 1% of cases were attributable to a history of CRC in FDR. Overall, the PAFs for advanced CRNs were higher than those for nonadvanced CRNs, and PAFs in males were higher than those in females, with the exception of physical inactivity and BMI.

Although CRC has been thought to be less common in Asia compared to Western countries, there is an increasing trend for CRC incidence in China [1, 23, 24]. In our study, an increased detection rate was observed for both nonadvanced and advanced neoplasm from the first to third round of screening in Tianjin, comparable to the trend of China. In addition, Zhao et al. [25] suggested that adherence to colonoscopy in the first round (2012–2014) was lower than that in the second round (2015–2017), which could partly contribute to the increased detection rate.

With regard to the percent with positive family history in FDR (7.8%), it was lower than that from most western countries, but was comparable with or even higher than other reports from China (2.7%-5.9%) [26, 27], with some studies only reporting an overall positive rate of high-risk assessment questionnaire, ranging from 3.3% to 15.0% [4, 28–30]. This could be partly caused by the wide geographical, economic and racial variation in in the pattern of cancer [2, 31, 32], which might be associates with multiple factors, such as genetic, lifestyle, and environmental factors.

In the present analysis, only a small proportion of CRNs were attributable to a history of CRC in FDR. Previous studies assessing the attributable risk of CRC conducted in China were limited, and a family history of CRC has not been considered before [15–17]. Even in studies from other countries, family history was rarely taken into account to estimate the PAFs of CRC [9, 11, 12, 33]. A study from Germany suggested that only 2% of neoplastic polyps were attributable to a history of CRC in FDR, lower than 8% to smoking in their population [10], which was in keeping with our results. A meta-analysis of 9.28 million individuals revealed that the increased risk by family history of CRC was significantly lower in older individuals (RR: 3.29 for < 40 years vs. 1.42 for ≥ 40 years, *p* = 0.017) [34]. Thus, this low contribution of family history could be influenced by the older cohort used in the present study, with 99.9% of participants aged ≥ 40 years. Moreover, as suggested by Hoffmeister et al., even by accounting for higher risks for CRC (RR = 2.25), the fraction with advanced CRN attributable to family history in FDR (9%) would not exceed that attributable to smoking [10].

Consistent with previous studies, current smoking had a detrimental effect on the development of CRN [35–38]. Our findings showed a higher proportion of CRNs attributable to current smoking in males than in females, which was consistent with previous studies in Asian countries, such as Japan (29.7% vs. 5.0%) [11] and Korea (9.2% vs. 1.0% for colon; 21.8% vs. 1.7% for rectum) [12], and two studies conducted in China (8.4% vs. 0.4%; 7.9% vs. 0.8%, respectively) [15, 17]. However, this sex-based difference was smaller in Europe (8.3% in males vs. 5.9% in females) than in Asia [39], despite the opposite trend being observed in the UK (7% in males vs. 10% in females) [40] and a PAF as high as 12% in Norwegian females [41]. This discrepancy may be partly explained by the fact that in major tobacco-related cancers in Europe, incidence rates have fallen in males and risen in females [42]. In addition, the relative risks for smoking in all types of cancers remained similar among China, Korea and Japan, but it appeared to be much lower than those reported in Western countries [43], and this huge geographical difference was especially pronounced in lung cancer [44]. Thus, it is essential to evaluate the PAFs in each country, and the prevention strategy should also be country specific.

Alcohol consumption has been widely proven to be associated with an increased risk of CRC. Due to the much lower prevalence of alcohol consumption among women in China [45], PAFs in females were lower than those in males, which is consistent with most previous studies from other countries [11, 12, 15, 46, 47].

The prevalence of overweight and obesity has increased markedly in the Chinese population over the past decade [48]. The PAFs of CRC by overweight/obesity were 4.0%-5.3%, reported from China ten years ago [15, 16], while it increased to approximately 8% in the present study, in keeping with the increased prevalence of obesity. There also exists a geographical difference in BMI between Asia and Western countries, which potentially results from the different patterns of diet and lifestyle. For instance, most of the PAFs for obesity reported from Asia, including Japan [11], Korea [12, 49] and China [15, 16], were less than 10% regardless of sex, while those in the European population tended to be higher [46, 50]. Physical inactivity tended to contribute only a relatively small portion in nonadvanced CRN but appeared to be important in advanced CRN. Previous studies showed a wide range of PAFs physical inactivity, from 0.3% to 8.9%, partly due to different definitions of physical inactivity [11, 12, 15, 46].

The impact of high BMI between males and females has not been as consistent. Some studies showed a higher PAF of BMI in females [11, 15, 51], as did the present study, while others suggested a higher or similar PAF in males [46, 49, 50]. A study based on over one hundred thousand Asians revealed that a high BMI is associated with an increased risk of obesity-related cancer, including CRC, in Asian women, with a PAF of 6.7%, while that in men was -0.2% [52]. However, a meta-analysis of 56 studies found a stronger association between BMI and the risk of CRC for men than women [53]. One possible explanation was the relatively low prevalence of smoking and alcohol drinking in females, while those were major attributable factors in males. Hence, the relative proportion of cases attributable to BMI in females may appear to be higher than that in males. In addition, several studies have proven that BMI may be more strongly associated with CRC for, or perhaps even limited to, premenopausal women but not among older women [54, 55], partly due to the changes in hormones critical for carcinogenesis. Thus, further research is needed to determine the precise mechanisms of BMI in males and females.

Nonadvanced and advanced neoplasm were both considered in the present study. With respect to potential implications for the design of CRC screening programs, advanced CRN is the most relevant outcome. However, it would be more reassuring and convincing if the effect of risk factors was consistent for nonadvanced and advanced neoplasm because both are considered to be part of the multistage model of colorectal carcinogenesis [56].

This study had some limitations. First, due to its observational design, this study is prone to confounding. Second, the exposures were based on self-reports and might include some misclassification. Third, the data were collected in Tianjin, China, and were not applicable to represent the whole country. Fourth, some other potential lifestyle risk factors, such as the intake of fruit, vegetable and red and processed meat, were not included. Fifth, people who had a first-degree relative with CRC were more inclined to take part in the screening and colonoscopy, which might reduce the contribution of family history. However, there are several strengths of this study, and the major one is the population-based design in a real screening setting, with strict standards applied to ensure the quality of data. Our findings could provide practical means for developing guidelines of CRC prevention and control in China.

## Conclusions

We found that 29% of advanced CRNs in males and 13.8% in females were attributable to modifiable lifestyle factors, while only less than 1% of those were attributable to a family history of CRC in FDR. Smoking is currently the main contributor to CRN in males and that was high BMI in females. Overall, modifiable lifestyle factors, including smoking, alcohol consumption, physical activity and BMI, have a larger contribution to CRN than family history. Our findings provide a valuable quantitative appraisal of the impact of different factors in CRNs, which is helpful in prioritizing cancer prevention and control strategies in China.

## Supplementary Information

**Additional file 1:** **Table S1.** Association of selected risk factors with colorectal neoplasms stratified by sex.

## Data Availability

The datasets analysed during the current study are not publicly available due to limitations of ethical approval involving the patient data and anonymity but are available from the corresponding author on reasonable request.

## Abbreviations

PAF: Population attributable fraction
CRN: Colorectal neoplasm
OR: Odd ratio
CI: Confidence interval
BMI: Body mass index
CRC: Colorectal cancer
FDR: First-degree relative
FOBT: Faecal occult blood test
