# Supplementary material for: Modifiable lifestyle factors have a larger contribution to colorectal neoplasms than family history
Source: BMC Cancer. 2022 Oct 7;22:1051. doi: 10.1186/s12885-022-10141-1 (PMC9547467; doi:10.1186/s12885-022-10141-1)
Supplement: Supplementary file 1 — Additional file 1: Table S1. Association of selected risk factors with colorectal neoplasms stratified by sex. [file 12885_2022_10141_MOESM1_ESM.docx]

| **Table S1** Association of selected risk factors with colorectal neoplasms stratified by sex | | | | | | |
| --- | --- | --- | --- | --- | --- | --- |
|  | Male | | | Female | | |
|  | Number of cases with risk factor (%) | Adjusted OR^a^(95% CI) | p | Number of cases with risk factor (%) | Adjusted OR^a^(95% CI) | p |
| **For nonadvanced CRN** | | | | | | |
| Smoking status (vs. never) | | | | | | |
| Former | 743 (7.6) | 0.95 (0.85-1.06) | 0.349 | 110 (1.4) | 1.13 (0.88-1.46) | 0.330 |
| Current |  |  |  |  |  |  |
| <15 cigarettes/day | 1401 (14.3) | 1.26 (1.15-1.38) | < 0.001 | 297 (3.7) | 1.57 (1.34-1.85) | < 0.001 |
| ≥15 cigarettes/day | 2083 (21.3) | 1.34 (1.24-1.45) | < 0.001 | 333 (4.1) | 2.13 (1.80-2.51) | < 0.001 |
| Alcohol consumption (vs. never) | | | | | | |
| <34 g/day | 1498 (15.3) | 1.01 (0.88-1.15) | 0.882 | 62 (0.8) | 1.18 (0.76-1.85) | 0.462 |
| ≥34 g/day | 692 (7.1) | 1.16 (1.07-1.27) | < 0.001 | 26 (0.3) | 1.24 (0.76-2.01) | 0.395 |
| Physical activity (vs. regular activity) | | | | | | |
| Physical inactivity | 5241 (53.5) | 1.01 (0.95-1.07) | 0.742 | 4512 (56.2) | 1.01 (0.96-1.07) | 0.639 |
| BMI, kg/m^2^ (vs. <25) | | | | | | |
| 25-29.9 | 3916 (40.0) | 1.12 (1.06-1.19) | < 0.001 | 2916 (36.3) | 1.19 (1.12-1.26) | < 0.001 |
| ≥30 | 624 (6.4) | 1.37 (1.20-1.56) | < 0.001 | 630 (7.8) | 1.38 (1.23-1.54) | < 0.001 |
| Family history of CRC in FDR (vs. no) | | | | | | |
| Yes | 757 (7.7) | 1.15 (1.03-1.28) | 0.015 | 707 (8.8) | 1.09 (0.99-1.21) | 0.076 |
| **For advanced CRN** | | | | | | |
| Smoking status (vs. never) | | | | | | |
| Former | 156 (7.3) | 0.83 (0.69-1.01) | 0.058 | 22 (1.8) | 1.42 (0.89-2.25) | 0.142 |
| Current |  |  |  |  |  |  |
| <15 cigarettes/day | 365 (17.2) | 1.47 (1.27-1.69) | < 0.001 | 67 (5.3) | 2.19 (1.65-2.91) | < 0.001 |
| ≥15 cigarettes/day | 456 (21.4) | 1.36 (1.19-1.56) | < 0.001 | 45 (3.6) | 1.89 (1.35-2.65) | < 0.001 |
| Alcohol consumption (vs. never) | | | | | | |
| <34 g/day | 411 (19.3) | 1.27 (1.03-1.55) | 0.023 | 5 (0.4) | 0.93 (0.36-2.40) | 0.885 |
| ≥34 g/day | 163 (7.7) | 1.44 (1.26-1.65) | < 0.001 | 7 (0.6) | 0.23 (0.03-1.68) | 0.146 |
| Physical activity (vs. regular activity) | | | | | | |
| Physical inactivity | 1089 (51.2) | 1.06 (0.96-1.17) | 0.267 | 697 (55.5) | 1.11 (0.98-1.26) | 0.095 |
| BMI, kg/m^2^ (vs. <25) | | | | | | |
| 25-29.9 | 869 (40.9) | 1.15 (1.04-1.27) | 0.007 | 457 (36.4) | 1.20 (1.05-1.36) | 0.006 |
| ≥30 | 148 (7.0) | 1.48 (1.20-1.81) | < 0.001 | 112 (8.9) | 1.53 (1.23-1.90) | < 0.001 |
| Family history of CRC in FDR (vs. no) | | | | | | |
| Yes | 151 (7.1) | 1.10 (0.91-1.33) | 0.344 | 92 (7.3) | 0.91 (0.73-1.14) | 0.432 |
| CRN, colorectal neoplasm; OR, Odds ratio; CI, confidence interval; BMI, body mass index; CRC, colorectal cancer; FDR, first-degree relative.  ^a^ ORs were adjusted for age at colonoscopy, educational level, marital status, smoking status, alcohol intake, physical activity, BMI, family history of CRC in first-degree relative and year of colonoscopy. | | | | | | |
